# Psychometric Properties of the Greek Apathy Evaluation Scale Clinician Version (AES-C) in MCI Patients and Cognitively Healthy Older Adults

**DOI:** 10.3390/bs16040498

**Published:** 2026-03-27

**Authors:** Mary Keramida, Magda Tsolaki, Eleni Poptsi, Moses Gialaouzidis, Mara Gkioka

**Affiliations:** 1 Department of Psychology, CITY College, University of York Europe Campus, 54626 Thessaloniki, Greece; 2 Greek Association of Alzheimer’s Disease and Related Disorders, 54643 Thessaloniki, Greece; 3 Laboratory of Neurodegenerative Diseases, Center for Interdisciplinary Research and Innovation, Aristotle University of Thessaloniki (CIRI-AUTh), 54124 Thessaloniki, Greece; 4 1st Department of Neurology, Medical School, Aristotle University of Thessaloniki, 54124 Thessaloniki, Greece; 5 Department of Psychology, University of West Macedonia, 53100 Thessaloniki, Greece; 6 Institute of Applied Biosciences, Centre of Research and Technology, 57001 Thessaloniki, Greece

**Keywords:** apathy, psychometrics, cognition, dementia

## Abstract

Apathy is a neuropsychiatric symptom that is present in various disorders, including dementia and Mild Cognitive Impairment (MCI). Patients with MCI who exhibit symptoms of apathy are at a higher risk of progressing to dementia compared to those with depressive symptoms. The aim of the present study was to investigate the psychometric properties of the clinician-rated version of the Apathy Evaluation Scale (AES-C) in a Greek sample of MCI patients and healthy older adults. The translation and adaptation of the scale were conducted using the forward–backward method. The final sample consisted of 100 participants, 14 men (*n* = 14) and 86 women (*n* = 86), with a mean age of 72 years. Participants were administered the translated and adapted version of the AES-C, as well as the Greek version of the Beck Depression Inventory. In terms of reliability, Cronbach’s alpha was found to be high (*α* = 0.91), indicating excellent internal consistency. Confirmatory Factor Analysis (CFA) revealed a one-factor solution with a very good model fit (RMSEA = 0.018, CFI = 0.985, TLI = 0.983, SRMR = 0.076). The AES-C can serve as an important addition to neuropsychological assessment for detecting apathy symptoms in patients with MCI, thereby contributing to the early prognosis of dementia.

## 1. Introduction

Dementia is a neurocognitive disorder characterized by a progressive decline in cognitive functions such as memory, language, and executive functioning, which significantly interferes with daily living and independence. Globally, dementia represents a major public health challenge due to population aging and increasing life expectancy. According to the World Health Organization (2021), approximately 57 million people were living with dementia worldwide in 2021, with nearly 10 million new cases diagnosed annually, and this number is expected to rise to 152.8 million by 2050 (Nichols et al., 2022). In Greece, epidemiological studies estimate that around 196,000 individuals currently live with dementia, a number expected to increase to approximately 356,000 by 2050 (Skamagka & Tsolaki, 2025).

Alongside the cognitive and functional impairments of dementia, patients often experience behavioral and psychological symptoms (BPSDs), such as anxiety, depression, apathy, delusions, hallucinations, and sleep disturbances, which further impact quality of life (Connors et al., 2023). Among the neuropsychiatric symptoms of dementia, apathy and depression often coincide and are frequent among BPSDs. A large longitudinal study by Grossman et al. (2021) reported a cumulative prevalence of apathy of about 48% in MCI (Clinical Dementia Rating CDR 0.5), ~74% in mild dementia (CDR 1.0) and ~82% in more moderate dementia (CDR 2.0). In general, apathy refers to a lack of motivation for daily activities, the need for others to execute activities, flat emotion and loss of interest in personal matters that would normally trigger an emotional response (Marin et al., 1991).

Apathy differs from depression as it is mainly characterized by lack of motivation. Although lack of motivation is also a symptom of depression, apathy syndrome does not involve thoughts of hopelessness, sadness or guilt (Leung et al., 2001). Apathy and depression should be distinguished, since understanding the prevalence, impact, and differentiation of these symptoms is essential for effective clinical management and care planning. For example, treatments effective for depression (e.g., SSRIs) often fail to improve apathy and may even worsen motivational deficits in some cases (Starkstein & Leentjens, 2008). Furthermore, based on the literature, most patients with dementia and apathy also presented depression, but less than one-third of patients with depression had comorbidity with apathy (Starkstein et al., 2005). This finding suggests that apathy and depression are two distinct constructs that may sometimes overlap. Moreover, Vicini Chilovi et al. (2009) found that among MCI patients categorized based on comorbid symptoms of apathy or depression, those with apathy were at a greater risk of developing dementia than those with depression alone. Therefore, early detection of apathy symptoms could be used as an important indicator of dementia progression in MCI patients.

Apathy may further intensify caregivers’ psychological distress and represent a considerable challenge in their caregiving responsibilities (Nobis & Husain, 2018; van Dalen et al., 2018). BPSDs are considered a primary reason for institutionalization (Azermai et al., 2013). Additionally, it is common for people with the behavioral variant of frontotemporal dementia who experience symptoms such as apathy to be misdiagnosed with a psychiatric disorder (Ducharme et al., 2015). Overall, delays in diagnosis, and consequently in referring patients for appropriate interventions, are common in dementia care (Geddes et al., 2020). These findings underscore the importance of early identification of behavioral symptoms of dementia, such as apathy, to optimize patient outcomes and enhance quality of life.

### 1.1. Existing Measures of Apathy

Apathy has often been assessed using multidimensional instruments designed to capture a range of neuropsychiatric symptoms. A widely used tool to assess neuropsychiatric symptoms in dementia patients is the Neuropsychiatric Inventory (NPI) (Cummings, 1997). NPI measures 12 domains: (1) agitation/aggression, (2) irritability/lability, (3) depression/dysphoria, (4) anxiety, (5) elation/euphoria, (6) apathy/indifference, (7) delusions, (8) hallucinations, (9) disinhibition, (10) aberrant motor behavior, (11) sleep, and (12) appetite (Cummings, 1997). NPI is considered a valid and reliable measure for use in both research and clinical settings, and it has been translated for the Greek population (Politis et al., 2004). Although NPI provides very useful information about the neuropsychiatric symptoms in dementia patients, it is not exclusively a scale for measuring apathy. Additionally, the responses are provided by the caregiver and not directly from the patient.

The need for more tailored scales specifically to assess apathy in dementia led to the development of tools like the Dementia Apathy Review and Rating (DAIR) (Strauss & Sperry, 2002). DAIR is a 16-item, caregiver-administered interview that produces a single-factor structure. It has demonstrated excellent internal consistency (*α* = 0.89) and high test–retest reliability (*r* = 0.85), indicating stable results over time. Apathy assessed through the DAIR has been associated with cognitive deficits rather than depression, supporting its discriminant validity (Strauss & Sperry, 2002). Similarly, the Dementia Apathy Scale (DAS) (Radakovic & Abrahams, 2014) is also administered to caregivers and uses informant input to assess apathy. Comprising 24 items, the DAS shows adequate internal consistency (*α* = 0.79) and assesses three factors: emotional blunting, initiation, and social interest.

To overcome the limitation of relying solely on informant perspectives, the Apathy Inventory (AI) (Robert et al., 2002) was developed to integrate data from caregivers, patients, and clinicians. This instrument includes nine items assessing three domains: emotional blunting, lack of initiative, and lack of interest. The informant version demonstrated satisfactory internal consistency (*α* = 0.84); however, the psychometric properties of the patient-rated version were not reported in the original validation (Robert et al., 2002). AI has been validated in both Alzheimer’s disease (AD) and Mild Cognitive Impairment (MCI), offering a more comprehensive assessment of apathy across multiple informants.

Among apathy measures, the Apathy Evaluation Scale (AES) (Marin et al., 1991) remains one of the most psychometrically robust and widely used tools. The AES has three versions: self-rated (AES-S), informant-rated (AES-I), and clinician-rated (AES-C), comprising 18 items rated on a 4-point Likert scale. AES-C demonstrates strong internal consistency (*α* = 0.90) and test–retest reliability (*r* = 0.88). The scale effectively differentiates apathy from depression, particularly in the clinician and self-rated forms (Marin et al., 1991).

Overall, existing apathy measures vary in their source of assessment and scope. Instruments such as DAIR and DAS rely on informant reports, providing valuable insights when patients have reduced awareness. The AI broadens the perspective by integrating caregiver, clinician, and patient input, whereas the AES offers distinct self, informant, and clinician-rated versions, allowing a holistic understanding of apathy. Among these, the AES-C stands out for its robust psychometric properties, including high internal consistency, test–retest reliability, and discriminant validity (Marin et al., 1991). Its unidimensional structure makes it particularly suitable for clinical applications requiring a nuanced understanding of apathy across emotional, behavioral, and cognitive domains. The AES has been validated in multiple linguistic and clinical contexts, including Swedish (Johansson et al., 2017), German (Lueken et al., 2007), Portuguese (Caeiro et al., 2012), Spanish (Martínez-Cao et al., 2022), Korean (Choi et al., 2020), and Italian (Furneri et al., 2021) populations, though it has not yet been validated for Greek speakers.

### 1.2. Aim of the Study

The present study aimed to translate the AES and examine the psychometric properties of the AES clinician version (AES-C) in a Greek sample including cognitively healthy older adults and individuals with MCI. The validation of this instrument is expected to facilitate the early diagnosis of apathy symptoms and support the identification of people at risk of progression from MCI to dementia. Specifically, the hypotheses of the present study are: (1) AES-C will have a unidimensional factor structure, (2) AES-C will demonstrate adequate internal consistency and (3) AES-C will demonstrate adequate convergence validity and be distinguishable from depression as measured by the BDI.

## 2. Materials and Methods

### 2.1. Design

A quantitative cross-sectional design was used to translate and validate the AES-C in Greek and to explore its psychometric properties.

### 2.2. Ethics

The sampling procedure took place at Alzheimer Hellas day care facilities after receiving ethical approval from the Greek Association of Alzheimer’s Disease and Related Disorders (GAARDD) Ethics Committee (protocol number: 96/11-01-2024). All procedures involving human participants were performed in accordance with the ethical standards of the bioethics committee and the 1964 Declaration of Helsinki and its subsequent revisions. Participants took part in the study voluntarily after signing informed consent. All participants’ data remained anonymous and confidential. Participants who wished to participate in the retest procedure were informed that their names would be kept until their results were matched and then deleted. The data were only accessible to the main researcher and were stored on a private password-protected computer.

### 2.3. Participants

Participants were cognitively healthy older adults and individuals diagnosed with MCI, recruited from the day care centers of Alzheimer’s Hellas, “Saint Hellen” and “Saint John”, in Thessaloniki, Greece, from October 2024 to October 2025. The sample size was determined based on the guidelines by Polit and Beck (2008), which suggest that a sample size of 5–10 participants per item is sufficient. Thus, a sample of 90 participants was deemed sufficient in proportion to the 18-item scale. The total sample consisted of 100 participants who took part voluntarily after signing an informed consent form. The study included both male (*n* = 14) and female (*n* = 86) participants ranging in age from 57 to 86 years, with a mean age of 72 years (*M* = 72, *SD* = 6.79). The total sample consisted of patients with MCI (*n* = 53) and cognitively healthy older adults (*n* = 47). Participants who wished to participate in the retest procedure were contacted within two weeks (*n* = 44) for a second assessment of AES-C.

### 2.4. Inclusion Criteria

All participants underwent an extended neuropsychological assessment for diagnostic purposes, which included the following tests: (a) the Mini-Mental State Examination (MMSE; Folstein et al., 1975; Tombaugh & McIntyre, 1992; Greek validation: Fountoulakis et al., 2000) and (b) the Montreal Cognitive Assessment (MoCA; Nasreddine et al., 2005; Greek validation: Lyrakos et al., 2020; Greek norms: Poptsi et al., 2019) for the assessment of global cognition; (c) the Functional Cognitive Assessment Scale (FUCAS; Kounti et al., 2006) for functional performance; (d) the Functional Rating Scale for Symptoms of Dementia (FRSSD; DeJong et al., 1989) for activities of daily living (caregiver evaluation); (e) the Rey Auditory Verbal Learning Test (RAVLT; Rey, 1958; Schmidt, 1996; Greek validation: Messinis et al., 2007) for verbal memory; (f) the Rey–Osterrieth Complex Figure Test (ROCFT; Osterrieth, 1944; Greek norms: Tsatali et al., 2022) for visual memory and visuoconstructive abilities; (g) the Verbal Fluency Test (FAS; Benton & Hamsher, 1976), the Trail Making Test Part B (Reitan, 1958; Reitan & Wolfson, 1985, Greek norms: Zalonis et al., 2008), and the Stroop Color-Word Test (Stroop, 1935; Greek norms: Zalonis et al., 2009) for executive functioning; and (h) the Digit Span Forward and Backward (Wechsler, 1981) for working memory. Finally, the Geriatric Depression Scale (GDS; Yesavage et al., 1983; Greek validation: Fountoulakis et al., 1999) was used to exclude participants with severe depressive symptoms (score ≥ 11).

The inclusion criteria for cognitively healthy older adults were: (a) a total score on the Mini-Mental State Examination (MMSE) greater than 28 and (b) no memory complaints. The Petersen criteria were applied for mild cognitive impairment (MCI) diagnosis (Petersen et al., 1999). The exclusion criteria comprised: (a) diagnosis of dementia based on the DSM-5 criteria (American Psychiatric Association, 2013); (b) uncontrolled psychiatric illness or affective disorder; (c) substance abuse or alcoholism; (d) history of traumatic brain injury; (e) brain tumors, encephalitis, or other neurological disorders; (f) cancer in the last 5 years, myocardial infarction in the last 6 months, history of stroke, presence of a pacemaker; (g) drug treatment with opioids, B12, folate, or thyroid medication; and (h) uncorrected sensory deficits.

### 2.5. Procedure

The procedure began with participants reading the participant information sheet, after which they were given the opportunity to ask the researcher questions. Afterwards, they signed the informed consent form to take part in the study. The clinician-rated version of the AES was administered to each participant individually, using a semi-structured interview (Marin et al., 1991). After that, participants were asked to complete the Beck Depression Inventory (BDI), which assesses depression symptoms, to test the discriminant validity of the scale. Participants’ demographic information (gender and age) and Mini-Mental State Examination scores were obtained from the patient database after they provided written consent.

### 2.6. Measures

#### 2.6.1. Apathy Evaluation Scale—Clinician Version

AES-C was developed by Marin et al. (1991) to assess apathy symptoms mainly in individuals with MCI and dementia. The scale has 18 items, which are assessed by a licensed psychologist or healthcare professional. The scale is scored on a 4-point Likert scale ranging from 1 = Not at all characteristic to 4 = A lot characteristic. For clinical purposes, apathy is considered a pathological construct; therefore, all items with a “+” sign need to be reverse-scored after administration as follows: 1 = 4, 2 = 3, 3 = 2 and 4 = 1. For example, a patient who scores 4 (A lot characteristic) on item 1, “He/She is interested in things”, is considered to exhibit no apathy; therefore, the reverse-scored value becomes 1. The only items not requiring reverse-scoring are items 6, 10 and 11. At the beginning of the assessment, the clinician asks the patient about their current interests and hobbies and keeps track of how many there are, the language used to describe them, and non-verbal cues. This introduction guides the clinician to score the scale combining both the participants’ responses and their own clinical observations. Overall, the administration procedure takes about 10–15 min, depending on the patient. Items marked with “Q” are quantifiable items, which require the clinician to further ask the participant about the frequency or context of an event and adjust the score accordingly. An example of such an item is “He/She has friends”. Items 3, 8, 13 and 16 are self-rated (SE) items; hence, the clinician scores them solely based on the participant’s answers. An example of a SE item is “Getting together with friends is important to him/her”. Lastly, items are coded as “B” (Behavioral), “C” (Cognitive) and “E” (Emotional). The scale was found to have excellent internal consistency (*α* = 0.90) and test–retest reliability (*r* = 0.88) (Marin et al., 1991). Furthermore, based on the original validation, it was found that the AES-C can discriminate apathy from depression, showing a weak-to-moderate correlation (*r* = 0.39) (Marin et al., 1991). The total score can range from 18 to 72, and for healthy individuals, scores typically range from 20 to 32 (Marin et al., 1991).

#### 2.6.2. Greek Adaptation of AES-C

Cultural adaptation was achieved using the forward–backward method in collaboration with bilingual partners of the study. Two translations from English to Greek were generated by independent translators, and the most conceptually valid one was selected by an expert in cognitive psychology. This version was then submitted for backward translation (from Greek to English) to a third independent reviewer to resolve the discrepancies. The backward translation was particularly useful to compare the translated version with the original one in terms of consistency and content meaning (Hambleton, 2001). The expert committee and the researcher applied final corrections based on feedback from five native Greek speakers who evaluated the final Greek version of AES-C based on comprehensibility. The feedback was overall positive, and participants reported that the items were easily understood and the language was straightforward. Only two participants reported mild difficulty in understanding item 8, so a bilingual translator refined the wording, and the final version was: “Το να φέρει μια δουλειά εις πέρας είναι σημαντικό για αυτόν/αυτήν.” (Appendix A).

#### 2.6.3. Beck Depression Inventory

The Beck Depression Inventory (BDI) is a well-known self-report measure of depression based on cognitive distortion theory (Beck et al., 1996). The BDI consists of 21 items, which represent 21 major symptoms of depression, scored on a 4-point Likert scale ranging from 0 to 3. For example, for the symptom of sadness, the participant is presented with four choices: 0 = “I do not feel sad.”, 1 = “I feel sad.”, 2 = “I am sad all the time and I can’t snap out of it.” and 3 = “I am so sad and unhappy that I cannot stand it.”. The patient has to choose the answer that best describes their mood during the past four weeks. The scale was originally developed by Beck et al. (1996), and several versions have been developed since then (BDI-I, BDI-IA, and BDI-II). The BDI has adequate psychometric properties and excellent internal consistency (α = 0.91) (Beck et al., 1996). For the present study, the second version of the scale (BDI-II) was used. The BDI-II was originally validated in a Greek population by Giannakou et al. (2013) and it showed very good internal consistency and test–retest reliability. Recently, the scale’s psychometric properties were examined in a Greek sample of middle-aged and older adults and the results revealed sufficient internal consistency (α = 0.87) (Economou et al., 2024).

### 2.7. Statistical Analysis

Statistical analyses were conducted using R (version 4.3.3) and RStudio (version 2024.12.0+467; Posit Software, PBC), utilizing relevant packages such as psych (version: 2.4.6.26; Revelle, 2018) for descriptive statistics, lavaan (version: 0.6-19; Rosseel, 2012) for the CFA and semPlot (version: 1.1.6; Epskamp, 2015) to visualize the path diagram. As for the reliability analysis, ltm (version: 1.2-0; Rizopoulos, 2006) was used to assess Cronbach’s alpha. Finally, ggplot2 (version: 3.5.1; Wickham, 2016) was used for data visualization where appropriate.

Descriptive statistics were computed using the mean and standard deviation. To assess the scale’s internal consistency, Cronbach’s alpha and McDonald’s omega were used. All values above 0.75 were considered adequate based on Christmann and Van Aelst (2006). Moreover, item–rest correlations were used to examine the scale’s internal consistency. Before proceeding with the Confirmatory Factor Analyses (CFA), Bartlett’s Test of Sphericity and the Kaiser–Meyer–Olkin statistic were used to assess the suitability of the data for factor analysis. CFA was performed using the robust Unweighted Least Squares (ULSMV) estimator, which is considered the most appropriate for ordinal data that do not follow a normal distribution (Kyriazos & Poga-Kyriazou, 2023). The fit indices used to assess the model fit included the Root Mean Square Error of Approximation (RMSEA), Standardized Root Mean Square Residual (SRMR), Tucker–Lewis Index (TLI), and Comparative Fit Index (CFI). The cut-off scores were determined based on previous literature (Hu & Bentler, 1999; Yuan et al., 2016): RMSEA < 0.08, SRMR < 0.08, TLI > 0.90 and CFI > 0.90.

Regarding the structural validity, Spearman’s correlation coefficients were used. Specifically, AES-C scores were compared with the BDI scores. There were five participants who did not complete the BDI; therefore, they were excluded from this analysis (*n* = 95). Finally, for the test–retest reliability, the scores of the first administration, denoted as T1, were matched to the scores of the second administration, denoted as T2 (obtained within two weeks), using Spearman’s correlation coefficient in a sample of 44 participants who agreed to take part in the retest procedure. The sample for the retest analyses was deemed sufficient based on Rea and Parker (2014).

## 3. Results

### 3.1. Descriptive Statistics

The scale’s descriptive statistics were calculated using the mean and standard deviation for each item (Table 1). Item–rest correlations ranged from 0.41 (item 8) to 0.73 (item 2), indicating an overall adequate level of internal consistency for the scale. The mean scores for each item ranged from 1.22 (*SD* = 0.48) (item 16) to 1.95 (*SD* = 0.78) (item 7).

### 3.2. Factor Analysis

**Figure 1:**
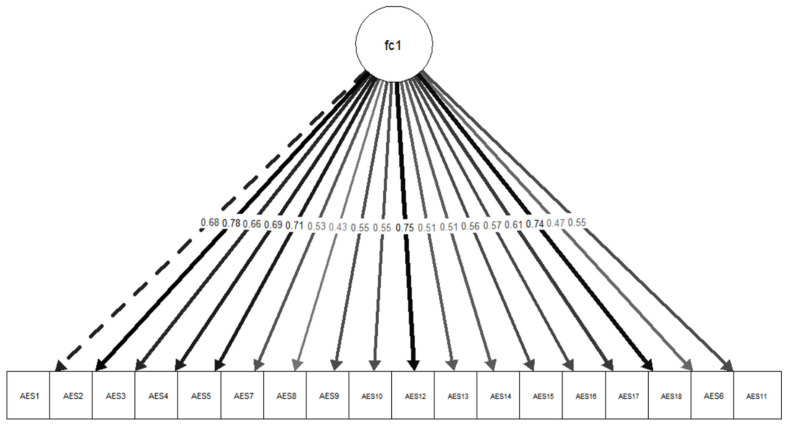
Path Diagram.

The total sample for the present analysis consisted of 100 participants (*N* = 100). Bartlett’s test of Sphericity demonstrated that the correlations were sufficiently strong to proceed with the CFA (*χ*^2^(153) = 814.14, *p* < 0.001). The Kaiser–Meyer–Olkin (KMO) statistic also indicated that the data are adequate for factor analysis (KMO = 0.87). The CFA was conducted using the ULSMV method, and model fit was evaluated using multiple fit indices. All items loaded onto a single factor, demonstrating adequate model fit: RMSEA (robust) = 0.018, CFI (robust) = 0.98, TLI (robust) = 0.98 and SRMR = 0.076. Factor loadings ranged from 0.43 (item 8) to 0.78 (item 2). Factor loadings for the one-factor model are presented in Table 2. Figure 1 provides a visual representation of the unidimensional factor structure. The unidimensional AES-C demonstrated excellent internal consistency, as evidenced by Cronbach’s alpha (*α* = 0.90) and McDonald’s omega (*ω* = 0.91).

### 3.3. Test–Retest Reliability

For the assessment of test–retest reliability, a subsample of 44 participants (*N* = 44) was selected from the original cohort and re-administered the scale within a two-week interval after they provided written consent. The subsample comprised 10 men (*n* = 10) and 34 women (*n* = 34), with a mean age of 72.45 years (*M* = 72.45, *SD* = 7.70), and included individuals with mild cognitive impairment (*n* = 24) as well as cognitively healthy adults (*n* = 20). Their scores on T1 and T2 were analyzed using Spearman’s rho, and the results showed a strong positive correlation (*r* = 0.89, *p* < 0.001). Cronbach’s alpha was calculated again for the second administration and the results demonstrated acceptable internal consistency (*α* = 0.79).

### 3.4. Structural Validity

The discriminant validity analysis utilized the MMSE scores of all participants (*N* = 100) and revealed a statistically significant, weak negative correlation of AES-C with MMSE scores (*r* = −0.21, *p* = 0.029), suggesting that lower cognitive functioning is associated with higher levels of apathy. Divergent validity analysis was conducted only in participants who completed the BDI (*N* = 95). The results indicated a moderate positive correlation between BDI scores and AES-C scores (*r* = 0.56, *p* < 0.001), suggesting partial overlap while supporting the distinction between depressive symptoms and apathy.

## 4. Discussion

The present study aimed to translate and evaluate the psychometric properties of the clinician-rated AES-C in a Greek sample of healthy older adults and individuals with MCI. The findings indicate that the Greek version of the AES-C demonstrates satisfactory psychometric characteristics, supporting its use as a valid and reliable instrument for assessing apathy in these populations.

Confirmatory factor analysis supported a one-factor model, suggesting that the AES-C captures a single overarching construct of apathy. This finding is consistent with the original validation by Marin et al. (1991), who reported that most items loaded primarily onto one factor (loadings ranging from 0.44 to 0.87), supporting the unidimensional conceptualization of apathy as a cohesive syndrome characterized by diminished motivation across behavioral, cognitive, and emotional domains.

Cross-cultural evidence further supports this structure. Several international adaptations, including the Italian (Furneri et al., 2021), Swedish (Johansson et al., 2017), and Korean (Choi et al., 2020) versions, also found that a one-factor solution explained the majority of the variance, indicating that the AES-C reliably captures a single latent construct across cultural settings. For instance, Furneri et al. (2021) found that the one-factor model accounted for a substantial proportion of variance in a mixed sample of older adults, MCI and AD patients. Similarly, Johansson et al. (2017) reported that the Swedish version retained a strong single-factor structure in a sample of community-dwelling older adults, while Choi et al. (2020) demonstrated a unidimensional structure in dementia patients using a short-form AES-C. However, some variability has been reported. The German version (Lueken et al., 2007) identified a two-factor model explaining 62.4% of the total variance, with the first factor encompassing problem awareness and motivation-related domains (54.7%) and the second reflecting social engagement and initiative. These differences may reflect cultural and clinical heterogeneity or variations in sample characteristics rather than fundamental structural divergence. Nevertheless, the predominance of unidimensional findings across studies supports the theoretical model proposed by Marin et al. (1991), suggesting that apathy represents a unified motivational construct. Accordingly, our findings support Hypothesis 1 and indicate that the Greek AES-C appears to maintain the same factor structure as the original and most international versions. Maintaining a unidimensional factor structure is advantageous when a scale assesses a single underlying construct, as it improves conceptual clarity, scoring simplicity, and overall interpretability (Reise et al., 2013).

The Greek AES-C demonstrated strong internal consistency and test–retest reliability, indicating that the instrument produces stable and consistent scores over time. This mirrors the excellent reliability of the original clinician-rated version (*α* = 0.90, *r* = 0.88) reported by Marin et al. (1991). Similarly high reliability indices have been found in multiple cultural validations: Italian (*α* = 0.91; Furneri et al., 2021), Swedish (α = 0.94; Johansson et al., 2017), Korean (*α* = 0.95; Choi et al., 2020), and German (*α* = 0.95; Lueken et al., 2007) versions all demonstrated excellent internal consistency, with test–retest reliability ranging from *r* = 0.71 to *r* = 0.81. These consistent results across studies suggest that the AES-C is a psychometrically robust instrument across diverse cultural or linguistic contexts. Thus, the present study supports the reliability of the AES-C for assessing apathy among Greek older adults and MCI patients, providing further support for its use in both research and clinical settings. Given that apathy often presents subtly in early neurocognitive disorders, having a stable and consistent measure is critical for identifying clinically meaningful changes over time (Radakovic & Abrahams, 2014). Therefore, the current findings support Hypothesis 2 and provide evidence that the Greek AES-C demonstrates excellent reliability, consistent with international validations.

A key aim of this study was to evaluate whether the Greek AES-C can effectively discriminate apathy from depression. Although apathy and depression share overlapping affective features, they remain conceptually and empirically distinct. Consistent with the original validation (Marin et al., 1991), which found a weak positive correlation with depression using the Zung Self-Rating Depression Scale (*r* = 0.35), the present study revealed a moderate correlation (*r* = 0.56) with the BDI, indicating partial but not complete overlap between the two constructs. These findings align with data by Starkstein et al. (2005), who emphasized that although apathy and depression may co-occur, they represent distinct syndromes with separate neurobiological substrates. Comparable results have been reported in international studies. The Italian version of the AES-C demonstrated a moderate positive association with depressive symptoms measured by the Hamilton Depression Scale (*r* = 0.45) but retained discriminant validity (Furneri et al., 2021). The Swedish version showed correlations ranging from *r* = 0.40 to r = 0.59 with depressive measures (Johansson et al., 2017), while the Korean adaptation exhibited a low correlation (*r* = 0.12) with depression, suggesting a clear distinction between the constructs (Choi et al., 2020). Together, these findings support the ability of the AES-C to discriminate apathy from depression across diverse clinical and cultural contexts.

Moreover, our results align with previous studies suggesting that the AES-C is less influenced by mood symptoms compared to other apathy measures (Levy & Dubois, 2006; Starkstein & Leentjens, 2008). This property enhances its clinical utility, particularly in older populations where depressive symptoms often co-occur with cognitive decline. Thus, hypothesis 3 is supported, suggesting that the Greek AES-C maintains strong discriminant validity. When compared to other apathy instruments, the AES-C retains its status as a gold standard due to its theoretical coherence and strong psychometric performance (Marin et al., 1991; Levy & Dubois, 2006). The AI (Robert et al., 2002) and the DAS (Radakovic & Abrahams, 2014) are also widely used but conceptualize apathy as a multidimensional construct. The AI comprises three domains: emotional blunting, lack of initiative, and lack of interest, while the DAS identifies four distinct apathy dimensions. Both scales demonstrate good reliability (AI: *α* = 0.84; DAS: *α* = 0.79–0.87), but their multidimensional nature contrasts with the unidimensional structure of the AES-C, which is theoretically rooted in Marin’s unified conceptualization of apathy as a singular motivational deficit.

Additionally, the clinician-administered nature of the AES-C provides a significant advantage. Guercio et al. (2015) demonstrated that clinician-rated apathy was a stronger predictor of progression from MCI to dementia compared to self-report or informant versions. Clinician ratings integrate observational data and professional judgment, reducing the influence of insight limitations that often affect self-assessments in cognitively impaired populations. Therefore, the psychometric robustness, unidimensional structure, and clinician-based administration of the AES-C collectively enhance its diagnostic and prognostic value in clinical settings.

### Limitations

The use of a convenience sample may limit the generalizability of the findings to populations with different sociodemographic or contextual characteristics. Nevertheless, convenience sampling is a widely used non-probabilistic method in clinical and psychometric research (Andrade, 2021). Therefore, the present findings should be interpreted as indicative rather than definitive, particularly with respect to underrepresented populations. Another limitation concerns the sample size used for the CFA. Although the model demonstrated satisfactory fit indices, future studies with larger samples would allow for further replication and strengthening of the factor structure. An additional limitation relates to the gender distribution of the sample, which consisted predominantly of females (86%). This is a common constraint in research focusing on dementia, since females (1) have higher survival rates and tend to be more aware when it comes to health-related issues (Mielke et al., 2014) and (2) are more susceptible due to genetic factors (Beam et al., 2018). To address potential gender differences, an exploratory Mann–Whitney U test was conducted, which indicated no statistically significant difference in apathy scores between women (*M* = 25.00, *SD* = 6.17) and men (*M* = 28.71, *SD* = 9.49) (U = 487.50, *p* = 0.256). Nevertheless, the relatively small number of male participants limits the interpretation of this comparison. Future studies should aim to recruit more gender-balanced samples in order to further examine potential gender differences and the measurement invariance of the AES-C. Lastly, it is worth noting that the scale was not tested in dementia patients but only in MCI and healthy older adults. The scale was validated only in individuals with MCI and healthy older adults, without inclusion of dementia patients, which limits applicability to the broader clinical population. The choice of the sample was based on the fact that MCI patients with apathy symptoms have a higher risk of progressing to dementia (Vicini Chilovi et al., 2009). Future studies should aim to validate the scale for other types of dementia (e.g., frontotemporal dementia).

## 5. Conclusions

In conclusion, the Greek version of the AES-C exhibits a unidimensional factor structure, excellent reliability, and strong discriminant validity. These results are consistent with findings from the original and international validations, supporting the AES-C as a psychometrically sound and cross-culturally robust tool for assessing apathy in older adults and MCI patients. The evidence reinforces the theoretical framework of apathy as a unified construct and highlights the clinical value of the AES-C for use in both research and diagnostic contexts.

## Figures and Tables

**Table 1:** Item-level descriptive statistics.

Item	Item–Rest Correlation	Mean	*SD*
AES1	0.636	1.410	0.552
AES2	0.737	1.510	0.628
AES3	0.625	1.340	0.590
AES4	0.665	1.390	0.584
AES5	0.665	1.290	0.478
AES6	0.436	1.660	0.807
AES7	0.510	1.950	0.783
AES8	0.414	1.160	0.368
AES9	0.517	1.520	0.643
AES10	0.535	1.210	0.518
AES11	0.529	1.400	0.532
AES12	0.720	1.580	0.713
AES13	0.494	1.310	0.581
AES14	0.494	1.290	0.498
AES15	0.540	1.350	0.557
AES16	0.542	1.220	0.484
AES17	0.572	1.400	0.651
AES18	0.700	1.530	0.731

**Table 2:** Factor Loadings.

Item	Estimate	Uniqueness
AES1	0.676	0.543
AES2	0.778	0.395
AES3	0.660	0.564
AES4	0.693	0.520
AES5	0.706	0.502
AES6	0.465	0.784
AES7	0.528	0.721
AES8	0.427	0.818
AES9	0.553	0.694
AES10	0.546	0.702
AES11	0.551	0.696
AES12	0.755	0.431
AES13	0.510	0.740
AES14	0.508	0.742
AES15	0.563	0.683
AES16	0.568	0.677
AES17	0.610	0.628
AES18	0.742	0.449

## Data Availability

The data presented in this study are available on request from the corresponding author due to privacy reasons.

## Appendix A

Ελληνική Κλίμακα Aξιολόγησης Aπάθειας (Έκδοση Κλινικού)

Oδηγίες: Βαθμολόγησε κάθε αντικείμενο βάσει συνέντευξης με τον εξεταζόμενο/την εξεταζόμενη. H συνέντευξη θα πρέπει να ξεκινήσει με μία περιγραφή των ενδιαφερόντων, των δραστηριοτήτων και της καθημερινής ρουτίνας του εξεταζόμενου/της εξεταζόμενης. Βασίστε την βαθμολογία σας τόσο σε λεκτικές όσο και σε μη λεκτικές πληροφορίες. Oι βαθμολογίες θα πρέπει να βασίζονται στις τελευταίες 4 εβδομάδες. Για κάθε αντικείμενο η βαθμολογία θα πρέπει να κρίνεται:

1. Καθόλου χαρακτηριστικό
2. Λίγο χαρακτηριστικό
3. Aρκετά χαρακτηριστικό
4. Πολύ χαρακτηριστικό

1. Ενδιαφέρεται για πράγματα. + C Q*
2. Κάνει πράγματα κατά την διάρκεια της ημέρας. + B Q
3. Το να ξεκινάει πράγματα μόνος του/μόνη της είναι σημαντικό για αυτόν/αυτήν. + C SE
4. Ενδιαφέρεται να έχει νέες εμπειρίες. + C Q
5. Ενδιαφέρεται να μαθαίνει νέα πράγματα. + C Q
6. Καταβάλλει λίγη προσπάθεια σε οτιδήποτε. − B
7. Προσεγγίζει την ζωή με ένταση. + E
8. Το να φέρει μια δουλειά εις πέρας είναι σημαντικό για αυτόν/αυτήν. + C SE
9. Ξοδεύει χρόνο κάνοντας πράγματα που τον/την ενδιαφέρουν. + B
10. Κάποιος πρέπει να του/της λέει τι να κάνει κάθε μέρα. − B
11. Aνησυχεί λιγότερο για τα προβλήματα του/της από ότι θα έπρεπε. − C
12. Έχει φίλους. + B Q
13. Το να συναντιέται με φίλους είναι σημαντικό για αυτόν/αυτήν. + C SE
14. Όταν κάτι καλό συμβαίνει, ενθουσιάζεται. + E
15. Έχει ακριβή κατανόηση των προβλημάτων του/της. + O
16. Το να κάνει πράγματα μέσα στη μέρα είναι σημαντικό για αυτόν/αυτήν. + C SE
17. Έχει πρωτοβουλία. + O
18. Έχει κίνητρο. + O

* Σημείωση: Aντικείμενα που έχουν θετική/αρνητική διατύπωση επισημαίνονται με +/−. Τύπος αντικειμένου: C = νοητικό (cognitive); B = συμπεριφορικό (behavior); E = συναισθηματικό (emotional); O = άλλο (other). Oι ορισμοί αντικειμένων αυτό-αξιολόγησης (SE) και ποσοτικοποιήσιμων (Q) αντικειμένων συζητούνται στις οδηγίες χορήγησης.

## References

[B1-behavsci-16-00498] American Psychiatric Association (2013). Diagnostic and statistical manual of mental disorders.

[B2-behavsci-16-00498] Andrade C. (2021). The inconvenient truth about convenience and purposive samples. Indian Journal of Psychological Medicine.

[B3-behavsci-16-00498] Azermai M., Kane J., Liperoti R., Tsolaki M., Landi F., Passmore A. P., Petrovic M., Cruz-Jentoft A. J. (2013). Management of behavioural and psychological symptoms of dementia: Belgium, Greece, Italy, United Kingdom. European Geriatric Medicine.

[B4-behavsci-16-00498] Beam C. R., Kaneshiro C., Jang J. Y., Reynolds C. A., Pedersen N. L., Gatz M. (2018). Differences between women and men in incidence rates of dementia and Alzheimer’s disease. Journal of Alzheimer’s Disease.

[B5-behavsci-16-00498] Beck A. T., Steer R. A., Brown G. (1996). Beck depression inventory–II. APA PsycTests.

[B6-behavsci-16-00498] Benton A. L., Hamsher K. D. (1976). Multilingual aphasia examination manual.

[B7-behavsci-16-00498] Caeiro L., Silva T., Ferro J., Pais-Ribeiro J., Figueira M. L. (2012). Metric properties of the Portuguese version of the apathy evaluation scale. Psicologia, Saúde & Doenças.

[B8-behavsci-16-00498] Choi Y. R., Lee Y. N., Jeong E., Chang S. O. (2020). Validity and reliability of the Korean version of the apathy evaluation dcale dhort form for patients with dementia. Journal of Korean Academy of Fundamentals of Nursing.

[B9-behavsci-16-00498] Christmann A., Van Aelst S. (2006). Robust estimation of Cronbach’s alpha. Journal of Multivariate Analysis.

[B10-behavsci-16-00498] Connors M. H., Teixeira-Pinto A., Ames D., Woodward M., Brodaty H. (2023). Distinguishing apathy and depression in dementia: A longitudinal study. Australian & NewZealand Journal of Psychiatry.

[B11-behavsci-16-00498] Cummings J. L. (1997). The neuropsychiatric inventory: Assessing psychopathology in dementia patients. Neurology.

[B12-behavsci-16-00498] DeJong R., Osterlund M., Roy G. (1989). Measurement of quality-of-life changes in patients with Alzheimer’s disease. Clinical Therapeutics.

[B13-behavsci-16-00498] Ducharme S., Price B. H., Larvie M., Dougherty D. D., Dickerson B. C. (2015). Clinical approach to the differential diagnosis between behavioral variant frontotemporal dementia and primary psychiatric disorders. American Journal of Psychiatry.

[B14-behavsci-16-00498] Economou A., Konsolaki E., Kalsi I. A., Psychountaki M. (2024). The beck depression inventory-II in community-dwelling middle-aged and older Greeks: Factor structure and demographic associations. SAGE Open.

[B15-behavsci-16-00498] Epskamp S. (2015). semPlot: Unified visualizations of structural equation models. Structural Equation Modeling: A Multidisciplinary Journal.

[B16-behavsci-16-00498] Folstein M. F., Folstein S. E., McHugh P. R. (1975). “Mini-mental state”: A practical method for grading the cognitive state of patients for the clinician. Journal of Psychiatric Research.

[B17-behavsci-16-00498] Fountoulakis K. N., Tsolaki M., Chantzi H., Kazis A. (2000). Mini mental state examination (MMSE): A validation study in Greece. American Journal of Alzheimer’s Disease & Other Dementias.

[B18-behavsci-16-00498] Fountoulakis K. N., Tsolaki M., Iacovides A., Yesavage J., O’Hara R., Kazis A., Ierodiakonou C. (1999). The validation of the geriatric depression scale (GDS) in Greece. Aging Clinical and Experimental Research.

[B19-behavsci-16-00498] Furneri G., Platania S., Privitera A., Martelli F., Smeriglio R., Razza G., Maci T., Castellano S., Drago F., Santagati M., Caponnetto P., Caraci F., Di Nuovo S. (2021). The apathy evaluation scale (AES-C): Psychometric properties and invariance of Italian version in mild cognitive impairment and Alzheimer’s disease. International Journal of Environmental Research and Public Health.

[B20-behavsci-16-00498] Geddes M. R., O’Connell M. E., Fisk J. D., Gauthier S., Camicioli R., Ismail Z., Alzheimer Society of Canada Task Force on Dementia Care Best Practices for COVID-19 (2020). Remote cognitive and behavioral assessment: Report of the alzheimer society of Canada task force on dementia care best practices for COVID-19. Alzheimer’s & Dementia: Diagnosis, Assessment & Disease Monitoring.

[B21-behavsci-16-00498] Giannakou M., Roussi P., Kosmides M. E., Kiosseoglou G., Adamopoulou A., Garyfallos G. (2013). Adaptation of the beck depression inventory-II to Greek population. Hellenic Journal of Psychology.

[B22-behavsci-16-00498] Grossman H. T., Sano M., Aloysi A., Elder G. A., Neugroschl J., Schimming C., Soleimani L., Zhu C. W. (2021). Prevalent, persistent, and impairing: Longitudinal course and impact of apathy in Alzheimer’s disease. Alzheimer’s & Dementia: Diagnosis, Assessment & Disease Monitoring.

[B23-behavsci-16-00498] Guercio B. J., Donovan N. J., Munro C. E., Aghjayan S. L., Wigman S. E., Locascio J. J., Amariglio R. E., Rentz D. M., Johnson K. A., Sperling R. A., Marshall G. A. (2015). The apathy evaluation scale: A comparison of subject, informant, and clinician report in cognitively normal elderly and mild cognitive impairment. Journal of Alzheimer’s Disease.

[B24-behavsci-16-00498] Hambleton R. K. (2001). The next generation of the ITC test translation and adaptation guidelines. European Journal of Psychological Assessment.

[B25-behavsci-16-00498] Hu L. T., Bentler P. M. (1999). Cutoff criteria for fit indexes in covariance structure analysis: Conventional criteria versus new alternatives. Structural Equation Modeling: A Multidisciplinary Journal.

[B26-behavsci-16-00498] Johansson M., Johansson P., Stomrud E., Hagell P., Hansson O. (2017). Psychometric testing of a Swedish version of the apathy evaluation scale. Nordic Journal of Psychiatry.

[B27-behavsci-16-00498] Kounti F., Tsolaki M., Kiosseoglou G. (2006). Functional cognitive assessment scale (FUCAS): A new scale to assess functional decline in dementia. International Journal of Geriatric Psychiatry.

[B28-behavsci-16-00498] Kyriazos T., Poga-Kyriazou M. (2023). Applied psychometrics: Estimator considerations in commonly encountered conditions in CFA, SEM, and EFA practice. Psychology.

[B29-behavsci-16-00498] Leung V. P., Lam L. C. W., Chiu H. F. K., Cummings J. L., Chen Q. L. (2001). Validation study of the Chinese version of the neuropsychiatric inventory (CNPI). International Journal of Geriatric Psychiatry.

[B30-behavsci-16-00498] Levy R., Dubois B. (2006). Apathy and the functional anatomy of the prefrontal cortex-basal ganglia circuits. Cerebral Cortex.

[B31-behavsci-16-00498] Lueken U., Seidl U., Völker L., Schweiger E., Kruse A., Schröder J. (2007). Development of a short version of the Apathy Evaluation Scale specifically adapted for demented nursing home residents. The American Journal of Geriatric Psychiatry.

[B32-behavsci-16-00498] Lyrakos G., Ypofandi M., Tzanne P. (2020). Psychometric and clinimetric properties of the montreal cognitive assessment (MoCA) in a Greek sample. European Psychiatry.

[B33-behavsci-16-00498] Marin R. S., Biedrzycki R. C., Firinciogullari S. (1991). Reliability and validity of the apathy evaluation scale. Psychiatry Research.

[B34-behavsci-16-00498] Martínez-Cao C., García-Álvarez L., Bobes-Bascarán T., De la Fuente-Tomas L., Fernández-Egea E., Velasco A., González-Blanco L., Zurrón-Madera P., Fonseca-Pedrero E., Sáiz-Martínez P. A., García-Portilla M. P., Bobes J. (2022). Validation of a European Spanish adaptation of the apathy evaluation scale-self-rated version (AES-S) in patients with schizophrenia. Revista de Psiquiatría y Salud Mental (English Edition).

[B35-behavsci-16-00498] Messinis L., Tsakona I., Malefaki S., Papathanasopoulos P. (2007). Normative data and discriminant validity of Rey’s suditory verbal learning test for the Greek adult population. Archives of Clinical Neuropsychology.

[B36-behavsci-16-00498] Mielke M. M., Vemuri P., Rocca W. A. (2014). Clinical epidemiology of Alzheimer’s disease: Assessing sex and gender differences. Clinical Epidemiology.

[B37-behavsci-16-00498] Nasreddine Z. S., Phillips N. A., Bédirian V., Charbonneau S., Whitehead V., Collin I., Cummings J. L., Chertkow H. (2005). The montreal cognitive assessment (MoCA): A brief screening tool for mild cognitive impairment. Journal of the American Geriatrics Society.

[B38-behavsci-16-00498] Nichols E., Steinmetz J. D., Vollset S. E., Fukutaki K., Chalek J., Abd-Allah F., Abdoli A., Abualhasan A., Abu-Gharbieh E., Akram T. T., Al Hamad H. (2022). Estimation of the global prevalence of dementia in 2019 and forecasted prevalence in 2050: An analysis for the global burden of disease study 2019. The Lancet Public Health.

[B39-behavsci-16-00498] Nobis L., Husain M. (2018). Apathy in Alzheimer’s disease. Current Opinion in Behavioral Sciences.

[B40-behavsci-16-00498] Osterrieth P. A. (1944). Le test de copie d’une figure complexe; contribution à l’étude de la perception et de la mémoire [Test of copying a complex figure; contribution to the study of perception and memory]. Archives de Psychologie.

[B41-behavsci-16-00498] Petersen R. C., Smith G. E., Waring S. C., Ivnik R. J., Tangalos E. G., Kokmen E. (1999). Mild cognitive impairment: Clinical characterization and outcome. Archives of Neurology.

[B42-behavsci-16-00498] Polit D. F., Beck C. T. (2008). Nursing research: Generating and assessing evidence for nursing practice.

[B43-behavsci-16-00498] Politis A. M., Mayer L. S., Passa M., Maillis A., Lyketsos C. G. (2004). Validity and reliability of the newly translated hellenic neuropsychiatric inventory (H-NPI) applied to Greek outpatients with Alzheimer’s disease: A study of disturbing behaviors among referrals to a memory clinic. International Journal of Geriatric Psychiatry.

[B44-behavsci-16-00498] Poptsi E., Moraitou D., Eleftheriou M., Kounti-Zafeiropoulou F., Papasozomenou C., Agogiatou C., Bakoglidou E., Batsila G., Liapi D., Markou N., Nikolaidou E., Ouzouni F., Soumpourou A., Vasiloglou M., Tsolaki M. (2019). Normative data for the montreal cognitive assessment in Greek older adults with subjective cognitive decline, mild cognitive impairment and dementia. Journal of Geriatric Psychiatry and Neurology.

[B45-behavsci-16-00498] Radakovic R., Abrahams S. (2014). Developing a new apathy measurement scale: Dimensional apathy scale. Psychiatry Research.

[B46-behavsci-16-00498] Rea L. M., Parker R. A. (2014). Designing and conducting survey research: A comprehensive guide.

[B47-behavsci-16-00498] Reise S. P., Bonifay W. E., Haviland M. G. (2013). Scoring and modeling psychological measures in the presence of multidimensionality. Journal of Personality Assessment.

[B48-behavsci-16-00498] Reitan R. M. (1958). Validity of the trail making test as an indicator of organic brain damage. Perceptual and Motor Skills.

[B49-behavsci-16-00498] Reitan R. M., Wolfson D. (1985). The halstead–Reitan neuropsychological test battery: Theory and clinical interpretation.

[B50-behavsci-16-00498] Revelle W. (2018). psych: Procedures for psychological, psychometric, and personality research.

[B51-behavsci-16-00498] Rey A. (1958). L’examen clinique en psychologie *[The clinical examination in psychology]*.

[B52-behavsci-16-00498] Rizopoulos D. (2006). ltm: An R package for latent variable modeling and item response analysis. Journal of Statistical Software.

[B53-behavsci-16-00498] Robert P. H., Clairet S., Benoit M., Koutaich J., Bertogliati C., Tible O., Caci H., Borg M., Brocker P., Bedoucha P. (2002). The apathy inventory: Assessment of apathy and awareness in Alzheimer’s disease, Parkinson’s disease and mild cognitive impairment. International Journal of Geriatric Psychiatry.

[B54-behavsci-16-00498] Rosseel Y. (2012). lavaan: An R Package for Structural Equation Modeling. Journal of Statistical Software.

[B55-behavsci-16-00498] Schmidt M. (1996). Rey auditory verbal learning test: A handbook.

[B56-behavsci-16-00498] Skamagka S., Tsolaki M. (2025). Evaluation of dementia-related knowledge: Investigating awareness and perceptions in the Greek general population. Journal of Aging and Rehabilitation.

[B57-behavsci-16-00498] Starkstein S. E., Ingram L., Garau M. L., Mizrahi R. (2005). On the overlap between apathy and depression in dementia. Journal of Neurology, Neurosurgery & Psychiatry.

[B58-behavsci-16-00498] Starkstein S. E., Leentjens A. F. (2008). The nosological position of apathy in clinical practice. Journal of Neurology, Neurosurgery & Psychiatry.

[B59-behavsci-16-00498] Strauss M. E., Sperry S. D. (2002). An informant-based assessment of apathy in Alzheimer disease. Cognitive and Behavioral Neurology.

[B60-behavsci-16-00498] Stroop J. R. (1935). Studies of interference in serial verbal reactions. Journal of Experimental Psychology.

[B61-behavsci-16-00498] Tombaugh T. N., McIntyre N. J. (1992). The mini-mental state examination: A comprehensive review. Journal of the American Geriatrics Society.

[B62-behavsci-16-00498] Tsatali M., Emmanouel A., Gialaouzidis M., Avdikou K., Stefanatos C., Diamantidou A., Kouroundi E., Messini C., Tsolaki M. (2022). Rey complex figure test (RCFT): Norms for the Greek older adult population. Applied Neuropsychology: Adult.

[B63-behavsci-16-00498] van Dalen J. W., van Wanrooij L. L., van Charante E. P. M., Brayne C., van Gool W. A., Richard E. (2018). Association of apathy with risk of incident dementia: A systematic review and meta-analysis. JAMA Psychiatry.

[B64-behavsci-16-00498] Vicini Chilovi B., Conti M., Zanetti M., Mazzù I., Rozzini L., Padovani A. (2009). Differential impact of apathy and depression in the development of dementia in mild cognitive impairment patients. Dementia and Geriatric Cognitive Disorders.

[B65-behavsci-16-00498] Wechsler D. (1981). WAIS–R manual: Wechsler adult intelligence scale–revised.

[B66-behavsci-16-00498] Wickham H. (2016). Getting started with ggplot2. ggplot2: Elegant graphics for data analysis.

[B67-behavsci-16-00498] World Health Organization (2021). Dementia fact sheet.

[B68-behavsci-16-00498] Yesavage J. A., Brink T. L., Rose T. L., Lum O., Huang V., Adey M., Leirer V. O. (1983). Development and validation of a geriatric depression screening scale: A preliminary report. Journal of Psychiatric Research.

[B69-behavsci-16-00498] Yuan K., Chan W., Marcoulides G. A., Bentler P. M. (2016). Assessing structural equation models by equivalence testing with adjusted ft indexes. Structural Equation Modeling: A Multidisciplinary Journal.

[B70-behavsci-16-00498] Zalonis I., Christidi F., Bonakis A., Kararizou E., Triantafyllou N., Paraskevas G., Kapaki E., Vasilopoulos D. (2009). The Stroop effect in Greek healthy population: Normative data for the stroop neuropsychological screening test. Archives of Clinical Neuropsychology.

[B71-behavsci-16-00498] Zalonis I., Kararizou E., Triantafyllou N. I., Kapaki E., Papageorgiou S., Sgouropoulos P. E. E. A., Vassilopoulos D. (2008). A normative study of the trail making test A and B in Greek adults. The Clinical Neuropsychologist.

